# Expanding the Reaction Space of Linkage-Specific Sialic Acid Derivatization

**DOI:** 10.3390/molecules24193617

**Published:** 2019-10-08

**Authors:** Tamas Pongracz, Manfred Wuhrer, Noortje de Haan

**Affiliations:** Center for Proteomics and Metabolomics, Leiden University Medical Center, 2333ZA Leiden, The Netherlands; t.pongracz@lumc.nl (T.P.); m.wuhrer@lumc.nl (M.W.)

**Keywords:** glycosylation, sialic acids, derivatization, isomer-specificity, catalysts, mass spectrometry, glycomics

## Abstract

The human glycome is characterized by a high degree of sialylation, affecting, amongst others, cell–cell interactions and protein half-life. An established method for the linkage isomer-specific characterization of *N*-glycan sialylation is based on the linkage-specific derivatization of sialylated glycoconjugates, inducing ethyl esterification of α2,6-linked sialic acids and lactonization of α2,3-linked sialic acids. While the carboxylic acid activator and nucleophile used in this reaction received extensive investigation, the role of the catalyst was never thoroughly explored. A frequently used catalyst for the linkage-specific esterification of sialic acids is 1-hydroxybenzotriazole (HOBt). Here, a systematic evaluation was performed of five HOBt alternatives in combination with 1-ethyl-3-(3-dimethylaminopropyl) carbodiimide (EDC) in ethanol for the linkage-specific derivatization of sialic acids. Derivatized glycans were analyzed by MALDI-TOF-MS and the catalyst performance was evaluated based on the completeness of the reactions and the linkage-specificity obtained. The use of both 6-Cl-HOBt and 6-CF_3_-HOBt resulted in high linkage-specificity and minimal byproduct formation, similar to the benchmark method using HOBt. Performing the reaction with these catalysts at neutral or acidic pH showed comparable efficiencies on both sialyllactose and complex-type *N*-glycans. The reported investigations resulted in an expansion of the reaction space for linkage-specific sialic acid derivatization.

## 1. Introduction

Protein glycosylation is one of the most abundant co- and post-translational modifications and has been reported as an important contributor to key physiological and pathological processes [1,2]. For example, it plays a major role in glycoprotein folding and solubility as well as in neoplastic transformations and metastasis [2,3,4,5]. Sialic acids (i.e., *N*-acetylneuraminic acids and *N*-glycolylneuraminic acids) commonly terminate glycan structures in vertebrates and exert diverse biological roles. They are involved in the mediation of cell–cell interactions, lectin binding, and microbial attachment [6]. Sialic acids are typically attached in either α2,3- or α2,6-linkage to a subterminal galactose, and quantitative changes in the occurrence of these linkages have been reported in cancer development and metastasis [6,7].

Matrix-assisted laser desorption/ionization time-of-flight mass spectrometry (MALDI-TOF-MS) is widely used for the compositional analysis of *N*-glycans [8,9,10]. However, the relative quantification of neutral and acidic sugars in a single measurement is impeded using this technique, as sialylated glycans are prone to the formation of multiple alkali metal adducts. Furthermore, the α-glycosidic bond between sialic acids and their adjacent monosaccharide is notorious for its instability during MALDI, resulting in a vast underestimation of sialylated species [10]. Various measures may be taken to avoid ionization biases and desialylation during MALDI, such as the use of a cold matrix [11], the protection of sialic acids with permethylation [8], or the esterification or amidation of the carboxyl groups [10,12].

The latter approach allows the differential modification of sialic acids with distinct glycosidic linkages, enabling their discrimination by MS. To obtain linkage-specific derivatives, a nucleophile (i.e., an alcohol or an amine) is customarily applied in the presence of a condensing reagent. A successful reaction involves the nucleophile to react with α2,6-linked sialic acids, while α2,3-linked sialic acids form a lactone [13,14].

Strategies in linkage-specific sialic acid derivatization are often derived from those used in peptide synthesis workflows [15]. Essentially, the reactions start by the activation of the carboxyl group using a coupling agent. This equips the carboxylic acid with a good leaving group (e.g., an active ester). Subsequently, the activated carboxyl group reacts with a catalyst followed by aminolysis in the presence of an amine, or ester formation in case alcohol is the available nucleophile [10,16]. The latter reaction may also happen intramolecularly, resulting in the formation of lactones. For peptide synthesis, 1-hydroxybenzotriazole (HOBt) is extensively used as a catalyst, enhancing the reaction efficiency and reducing side-product formation [15]. This catalyst has been introduced before for the linkage-specific derivatization of sialic acids, in combination with 1-ethyl-3-(3-dimethylaminopropyl) carbodiimide (EDC) in ethanol [13].

The choice of the nucleophile to amidate or esterify the α2,6-linked sialic acids has shown to be a crucial factor in the linkage-specificity of the derivatization [10,13,14,17,18,19,20,21,22,23] and condensing reagents as alternative to EDC have been evaluated [24]. However, the role of the catalyst, and the potential alternatives to improve the derivatization, is studied to a lesser extent.

**Figure 1:**
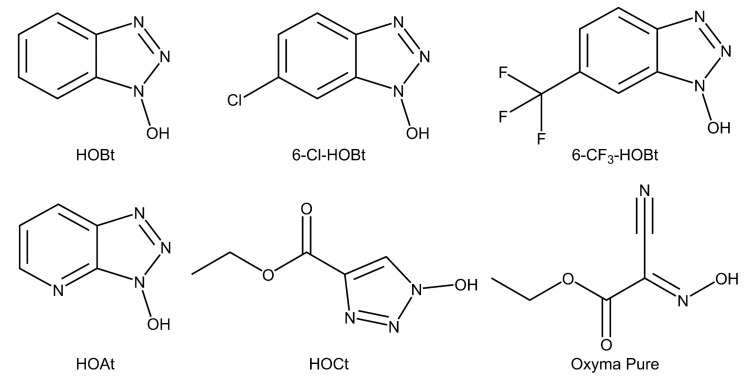
Chemical structure of the catalysts evaluated in this study. HOBt: 1-hydroxybenzotriazole, 6-Cl-HOBt: 6-chloro-1-hydroxybenzotriazole, 6-CF_3_-HOBt: 1-hydroxy-6-(trifluoromethyl)benzotriazole, HOAt: 3-hydroxytriazolo[4,5-b]pyridine, HOCt: ethyl 1-hydroxytriazole-4-carboxylate, Oxyma Pure: ethyl (2E)-2-cyano-2-hydroxyiminoacetate.

Here, we broaden the dimensions of the linkage-specific sialic acid derivatization toolbox by the systematic investigation of six catalysts: HOBt, 6-chloro-1-hydroxybenzotriazole (6-Cl-HOBt), 1-hydroxy-6-(trifluoromethyl)benzotriazole (6-CF_3_-HOBt), 3-hydroxytriazolo[4,5-b]pyridine (HOAt), ethyl 1-hydroxytriazole-4-carboxylate (HOCt), and ethyl (2*E*)-2-cyano-2-hydroxyiminoacetate (Oxyma Pure) (Figure 1) [13]. All catalysts were evaluated at different pH and temperature, while monitoring linkage-specificity and reaction completeness. For the final assessment of the chemicals, also toxicity and availability of the catalysts were taken into account (Table S1).

## 2. Results

**Figure 2:**
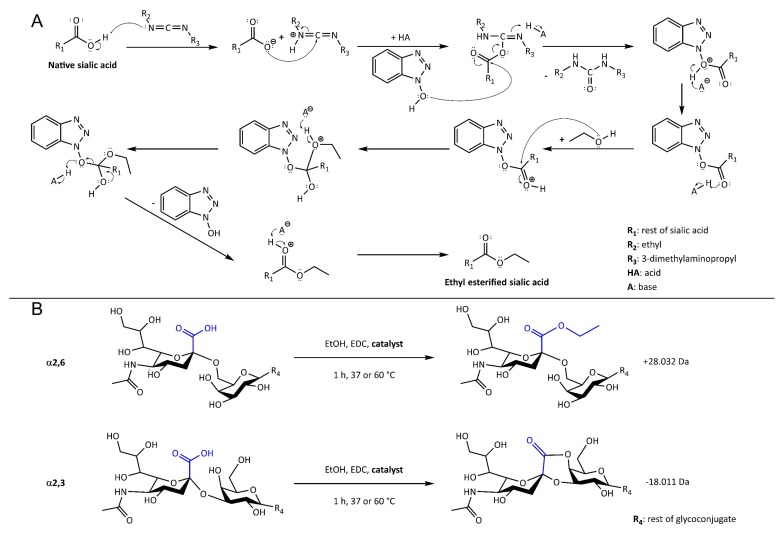
Reaction mechanism (**A**) and scheme (**B**) for the linkage-specific sialic acid derivatization using EDC, ethanol and the array of catalysts (in **A** exemplified by the catalyst HOBt (Figure 1)). The expected reaction products included ethyl ester formation on α2,6-linked sialic acids, thereby gaining +28.032 Da (**A** and **B**, **top**), while α2,3-linked sialic acids formed lactones under the same conditions (−18.011 Da; **B,** **bottom**). Misconversion leads to ethyl esterification on α2,3-linked sialic acids or lactone formation on α2,6-linked sialic acids with the same changes in mass as indicated above.

Six catalysts (Figure 1), in combination with EDC in ethanol, were evaluated for the sialic acid linkage-specific derivatization of sialylated glycoconjugates. The degree of ethyl ester (+28.032 Da) and lactone (−18.011 Da) formation was monitored for α2,6- and α2,3-linked sialic acids, respectively (Figure 2), along with non-specific reaction products originating from misconversion (i.e., ethyl esterification of α2,3-linked sialic acids or lactonization of α2,6-linked sialic acids). Incomplete reaction products were recognized as the unmodified mono- and disodiated adducts [M + Na]^+^ and [M − H + 2Na]^+^.

### 2.1. Evaluation of the Catalysts Using Sialyllactose Standards

**Figure 3:**
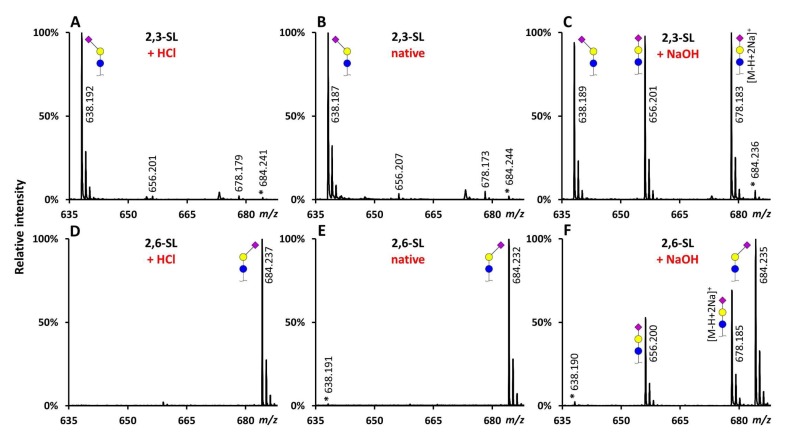
Representative MALDI-TOF-MS spectra of derivatized SL standards under different conditions using HOBt as catalyst. 2,3-SL (**A**–**C**) and 2,6-SL (**D**–**F**) incubated for 1 h at 37 °C in ethanol, with EDC and HOBt, at native (**B**,**E**) low (**A**,**D**) or high (**C**,**F**) pH. Lactonized reaction product: [M + Na]^+^ = 638.190 Da, ethyl esterified reaction product: [M + Na]^+^ = 684.232 Da. *: reaction product derived from misconversion of the standard (i.e., ethyl esterification of 2,3-SL or lactonization of 2,6-SL), standard impurity (reported purity: ≥98%), or a combination thereof. Symbols indicate the monosaccharide residues glucose (blue circle), galactose (yellow circle), and *N*-acetylneuraminic acid (purple diamond). In case of derivatized sialic acids, an α2,3-linkage is indicated by a left angle, an α2,6-linkage by a right angle, while non-derivatized sialic acids are depicted without an angle. Note: mass spectra are main peak normalized.

**Figure 4:**
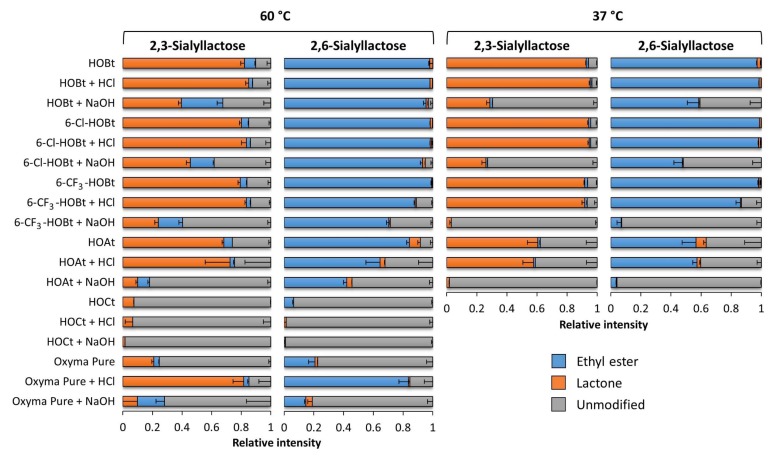
Ethyl esterification and lactonization, induced on the SL standards by the catalysts at different pH and temperatures. The catalysts were evaluated under native, acidic, and basic conditions at 60 °C (left panel) and 37 °C (right panel). All conditions were incubated for 1 h in ethanol, in the presence of EDC and one of the catalysts. The name of the catalyst without indication: native (pH 6), + HCl: acidified (pH 3), + NaOH: basified (pH 9). Shown are the average (stacked bars) and standard deviations (error bars) for triplicate measurements. Total area normalization was performed on modified and unmodified sialyllactose forms with the gross composition: hexose (H)2, N-acetylneuraminic acids (S)1 and their different sialic acid linkage variants H2L1 and H2E1 (lactonized α2,3-sialic acid: L; ethyl esterified α2,6-sialic acid: E). Additional information on spectra quality is included as Supplementary Materials (Figure S2) (Table S3).

All reaction conditions were applied on sialyllactose (SL) standards with known sialic acid linkages (2,3-SL and 2,6-SL). After a 1 h incubation at 37 °C at native conditions (pH 6), for 2,3-SL mainly the lactonized reaction product (average 92.7% ± standard deviation (SD) 0.3%) was observed at *m*/*z* 638.187, while for 2,6-SL the ethyl esterified product (97.2% ± 0.4%) was observed at *m*/*z* 684.232, as described before [13] (Figure 3B,E and Figure 4) (Table S2). The reactions at low pH (pH 3) showed comparable specificity (Figure 3A,D and Figure 4). In turn, the high pH (pH 9) conditions resulted in substantial amounts (>40%) of the underivatized byproducts ([M + Na]^+^ = 656.201 Da and [M − H + 2Na]^+^ = 678.183 Da) (Figure 3C,F and Figure 4).

The same reactions at elevated temperatures (as shown for 60 °C) resulted in similar specificities for 2,6-SL, while the degree of misconversion on 2,3-SL increased. In accordance, the degree of lactone formation on 2,3-SL decreased (82.2% ± 2.6% at pH 6) (Figure 4). Of note, under sub-optimal conditions (e.g., at 60 °C), the addition of an acidic modifier slightly improved the lactone formation on 2,3-SL (85.1% ± 2.0% at pH 3).

The use of the HOBt analogues 6-Cl-HOBt and 6-CF_3_-HOBt resulted in comparable yields for all reaction products as HOBt, when used at native conditions at 37 °C or 60 °C. While, 6-Cl-HOBt performed also similar to HOBt at low and high pH, 6-CF_3_-HOBt showed a substantial lower efficiency to catalyze the linkage-specific reactions at pH 3 for 2,6-SL (86.3% ± 3.0% ethyl ester formation at 37 °C) and at pH 9 for both linkage variants (>90% under modification at 37 °C) (Figure 4).

The catalysts HOAt resulted in a lower reaction efficiency and a higher degree of misconverted products as compared to HOBt and its 6-Cl- and 6-CF_3_-variants. The highest linkage-specificity using this catalyst was obtained under native conditions at 60 °C, resulting in 68.3% ± 1.2% lactone formation and 84.3% ± 1.8% ethyl ester formation on 2,3-SL and 2,6-SL, respectively. Moreover 5.7% ± 0.1% and 7.4% ± 1.8% misconversion was observed on 2,3-SL and 2,6-SL, respectively. HOCt resulted in >90% under modification at 60 °C, irrespective of the pH, and was not further investigated as a red precipitate was formed in the reaction mixture after addition of the sample. Although Oxyma Pure resulted in relatively high linkage-specificity at acidic conditions at 60 °C (81.7% ± 7.2% and 83.6% ± 6.3% for 2,3 and 2,6-SL, respectively), the use of this catalyst was compromised as byproducts were formed with ammonia. Amidated SL was observed at *m*/*z* 655.221 (−0.984 Da) giving rise to an overlapping isotopic pattern with the unmodified standard, as described earlier [13] (Figure S1), therefore Oxyma Pure was not investigated further.

### 2.2. Evaluation of the Catalysts Using Complex N-glycan Samples

**Figure 5:**
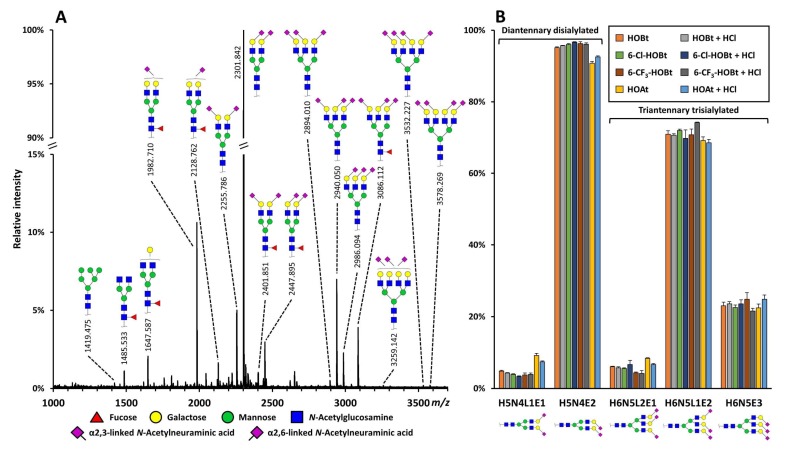
Representative MALDI-TOF-MS spectrum of derivatized *N*-glycans released from total plasma (TPNG) using 6-Cl-HOBt as catalyst at native conditions, and linkage-specificity of the reaction conditions as determined on a TPNG sample. (**A**) Mass spectrum showing relative intensities from *m*/*z* 1000 to 3700. Compositional assignments were based on accurate mass and isotopic pattern matching, structural assignments were based on literature and biosynthetic pathways. Except for the *N*-acetylneuraminic acids, glycosidic linkages were not determined. The assigned glycan signals had a signal-to-noise (S/N) > 9. Different sialic acid linkages are indicated by a left (α2,3) or right (α2,6) angle. (**B**) Relative ratio of di- and triantennary sialylated species with the gross composition hexose (H)5; *N*-acetylhexosamine (N)4; *N*-acetylneuraminic acids (S)2; and H6N5S3 and their different sialic acid linkage variants (lactonized α2,3-sialic acid: L; ethyl esterified α2,6-sialic acid: E) after incubation with the different catalysts under native and acidic pH at 37 °C. Note: the di- and triantennary glycans are total area normalized to 100% separately. Error bars represent SD for triplicate measurements.

The catalysts 6-Cl-HOBt, 6-CF_3_-HOBt, and HOAt were further evaluated for their performance on more complex *N*-glycans released from total plasma (TPNG) by comparing their results to the results obtained by using HOBt, as this catalyst has proven its high efficiency and linkage-specificity on complex-type *N*-glycans before [13]. Incubating the TPNG sample with any of these catalysts at 37 °C at native or acidic pH, resulted in the identification of high-mannose and complex-type *N*-glycans with varying degrees of fucosylation, galactosylation, bisection, and sialylation, as expected [25,26] (Figure 5A) (Table S4). The sample was characterized by a high degree of α2,6- and α2,3-linked sialylation, and the highest abundant glycan in the spectra was a diantennary disialylated specie occupied with two α2,6-linked sialic acids, which is in agreement with literature [25,26] (Figure 5A).

To further assess the linkage-specificity of the reactions using 6-Cl-HOBt, 6-CF_3_-HOBt, or HOAt, the relative ratios of different sialic acid linkage-variants of the di- and triantennary sialylated species with the gross compositions hexose (H)5, *N*-acetylhexosamine (N)4, *N*-acetylneuraminic acids (S)2, and H6N5S3 were compared to those obtained by using HOBt at native pH. The diantennary species included H5N4L1E1 and H5N4E2 (L: lactonized α2,3-sialic acid; E: ethyl esterified α2,6-sialic acid), the triantennary species included H6H5L2E1, H6N5L1E2, and H6N5E3. At both neutral and acidic pH, the use of 6-Cl-HOBt and 6-CF_3_-HOBt resulted in similar relative intensities for the di- and triantennary species as were observed with the use of HOBt (Figure 5B) (Table S5). On the other hand, by using HOAt as catalyst, the α2,6-linked sialylated species were underestimated. For example, the relative abundance of H5N4E2 after the reaction at pH 6 was 90.8% ± 0.2% or 96.1% ± 0.2% with the use of HOAt or HOBt, respectively (Figure 5B) (Table S5). All conditions showed an average relative standard deviation of <7.5% over all glycoforms in the triplicate measurements.

## 3. Discussion

Linkage-specific sialic acid derivatization for enhanced MS-based glycomics is often performed using the carboxylic acid activator EDC and the catalyst HOBt in ethanol [13,18,23,27,28]. While the activator, the nucleophile and the solvent in this reaction underwent thorough investigations before [13,14,17,18,19,20,21,22,23,27,29,30,31,32,33,34], no reports were made on the role of the catalyst used. Here we reported on the comparison of six catalysts for linkage-specific sialic acid derivatization, including three hydroxybenzotriazole (HOBt)-based derivatives (6-CF_3_-HOBt, 6-Cl-HOBt, and HOAt), one hydroxytriazole (HOCt), and an alternative non-hydroxy(benzo)triazole-based compound (Oxyma Pure). The performance of all catalysts was evaluated as compared to the use of HOBt, which has been shown to result in excellent sialic acid linkage-specificity in combination with EDC and ethanol before [13,35]. While the here evaluated catalysts were hardly studied in the context of sialic acid derivatization before, they were for the catalysis of peptide synthesis. In those studies, HOAt (with a nitrogen atom at the 7 position of the benzotriazole) was reported to perform superior to HOBt, due to the electron withdrawing effect of the nitrogen atom and its stabilizing effects on reaction intermediates [36]. Correspondingly, the 6-substitution of HOBt with an electron withdrawing group, was reported to enhance reactivity [37], and also HOCt has been reported as potent alternative for HOBt in peptide synthesis [38]. Ultimately, the use of Oxyma Pure was reported as a non-explosive alternative to HOBt(-analogues) [39,40], but with higher risk of acute toxicity. For the final selection of catalysts used in this report, commercial availability was also taken into account.

All tested catalysts showed to be able to induce some degree of linkage-specific sialic acid derivatization on the simple sialyllactose standards. Although, with the use of HOCt the derivatization rate obtained was below 10% for all conditions tested. In general, performing the reactions at a higher temperature improved the ethyl ester formation on α2,6-linked sialic acids. On the other hand, lactone formation was negatively affected using higher temperature—likely caused by the faster hydrolysis of lactones at higher temperatures—showing an increase of non-specific ethyl esterification as well as of unmodified species. Of note, non-linkage-specific byproducts on the sialyllactose standards may derive either from misconversion or from the correct conversion of standard impurities. With the current MALDI-MS approach it was not possible to separate these effects. However, standard impurity was assumed to be less than 1%, as this was the minimum percentage of “misconversed” byproduct observed using HOBt as catalyst. This was further supported by the reported purity of the standard of ≥ 98% by the vendor.

The addition of an acidic modifier did not substantially affect the derivatization yield obtained by using most of the catalysts, neither on the SL standards, nor on the complex-type *N*-glycans. A notable exception was for the use of Oxyma Pure, where a low pH dramatically increased the linkage-specific yield for both α2,3- and α2,6-linked sialic acids [12]. Importantly, while sialylated species are known to be instable under harsh acidic conditions [41], no signs of (partial) desialylation were observed for the conditions tested in this study. For example, this was indicated by the highly similar relative ratios between neutral and sialylated complex-type *N*-glycans of TPNG, observed for the use of acidic and neutral conditions. HOBt (analogue)-mediated reactions are acid catalyzed [13], which matched the current observation that high pH reaction conditions were overall not favorable for the derivatization of sialic acids. Interestingly, non-specific ethyl esterification of 2,3-SL was increased under basic conditions at higher temperatures, indicating that the reaction of α2,3-linked sialic acids with external nucleophiles likely occurs independent from the added catalyst at high pH, as was suggested for amidation reactions before [19,21].

HOBt, 6-Cl-HOBt, and 6-CF_3_-HOBt resulted in the highest derivatization efficiency and showed superior linkage-specificity over the other catalysts under the conditions evaluated. This indicated the direct replaceability of HOBt with 6-Cl-HOBt or 6-CF_3_-HOBt in methods using EDC and ethanol for linkage-specific sialic acid derivatization. Moreover, the limited efficiency of HOCt was likely due to its low solubility in ethanol and water. This effect may be eliminated by using another solvent, such as dimethylformamide. Finally, while the use of Oxyma Pure resulted in high linkage-specificity at acidic conditions, the use of this catalyst induced amidation with ammonia on the 2,6-SL. This is likely caused by an ammonia contamination of the chemical as reported before [13] and excluded the usability of this catalyst in the current study. Obtaining a non-contaminated version of this chemical from another source may result in an attractive non-explosive alternative to HOBt. No indications for the presence of such byproducts were observed when using the other catalysts.

Recent trends in linkage-specific sialic acid derivatization focus on two-step derivatization protocols to introduce stable amide derivatives on α2,3-linked sialic acids after initial lactone formation of these species [17,18,19,21,22]. Although the current study does not take into account such two-step protocols, the presented results are still of relevance for these workflows, as they all depend on the formation of lactones on α2,3-linked sialic acids in the first step of the reaction. The general effect of a catalyst in the amidation step (second step) of these reaction remains to be investigated [19].

In conclusion, we showed that the catalysts 6-Cl-HOBt and 6-CF_3_-HOBt are directly applicable in workflows using EDC and ethanol for linkage-specific sialic acid derivatization of simple and complex sialylated glycans, as alternative to HOBt. The different physico-chemical properties of these chemicals may additionally allow a broader flexibility in solvent selection, which in turn would allow the linkage-specific amidation or esterification of sialic acids in different workflows using filter plates and/or targeting intact glycoproteins. For example, the derivatization of intact glycoproteins immobilized on hydrophobic polyvinylidene fluoride membranes is currently hampered by the incompatibility of such membranes with high ethanol concentrations [42]. Increasing the chemical space for sialic acid derivatization by the inclusion of alternative catalysts, will be beneficial for both in-solution and solid-phase MS-based glyco(proteo)mic workflows in the future.

## 4. Materials and Methods

### 4.1. Chemicals, Reagents, and Enzymes

All materials and reagents used in this study were of analytical grade and purchased from commercial suppliers. Type I Ultrapure Water (UP) was produced by an ELGA Purelab Ultra system (Elga LabWater, High Wycombe, United Kingdom). Ethanol, sodium hydroxide (NaOH), sodium dodecyl sulphate (SDS), and trifluoroacetic acid (TFA), disodium hydrogen phosphate dihydrate (Na_2_HPO_4_∙2H_2_O), potassium dihydrogen phosphate (KH_2_PO_4_), and sodium chloride were purchased from Merck (Darmstadt, Germany). 1-Hydroxybenzotriazole (HOBt) hydrate, ethyl (2E)-2-cyano-2-hydroxyiminoacetate, hydrochloric acid (HCl), Nonidet P-40 (NP-40), and ortho-phosphoric acid were obtained from Sigma-Aldrich Chemie (Steinheim, Germany), while 1-ethyl-3-(3-dimethylaminopropyl) carbodiimide (EDC) hydrochloride was acquired from Fluorochem (Hadfield, UK). 1-Hydroxy-6-(trifluoromethyl)benzotriazole and ethyl 1-hydroxytriazole-4-carboxylate were purchased from Tokyo Chemical Industy Co, Ltd. (Tokyo, Japan), 6-chloro-1-hydroxybenzotriazole from Acros Organics (Geel, Belgium), while 1-hydroxy-7-azabenzotriazole was obtained from Toronto Research Chemicals Inc. (Toronto, Canada). HPLC Supra-gradient acetonitrile (ACN) originated from Biosolve BV (Valkenswaard, Netherlands), 2,5-dihydroxybenzoic acid (2,5-DHB) and Peptide Calibration Mix II from Bruker Daltonics (Bremen, Germany). Finally, recombinant peptide-*N*-glycosidase F (PNGase F) was purchased from Roche Diagnostics (Mannheim, Germany).

5× PBS was prepared by dissolving 40.50 g NaCl, 0.93 g KCl, 20.28 g Na_2_HPO_4_·7H_2_O, and 1.09 g KH_2_PO_4_ in 1000 mL UP water (pH 7.2). Acidic PBS (pH 5.6) was prepared by adding 68 µL of 85% orto-phosphoric acid to 9932 µL 5× PBS. The *N*-glycan release mixture was composed of 10 µL 4% NP-40, 10 µL acidic 5× PBS, and 1 µL PNGase F.

### 4.2. Samples

Commercially available SL standards with known linkage-specificity, namely 2,3-SL sodium salt and 2,6-SL sodium salt (both with purities higher than 98%) were obtained from Carbosynth (Compton, UK). Pooled plasma from twenty healthy human donors (Visucon-F Frozen Normal Control Plasma, citrated and 0.02 M HEPES buffered) originated from Affinity Biologicals (Ancaster, ON, Canada). SL standards were dissolved to a final concentration of 100 mg/mL in UP water, and the pooled plasma was used without dilution before further processing.

### 4.3. Preparation of Catalysts

Each of the six catalysts were individually dissolved in ethanol and used in a combination with EDC in a final concentration of 0.25 M of both the catalyst and EDC. All chemicals readily dissolved, with the exception of 6-Cl-HOBt and HOAt, which were ultrasonicated in the presence of EDC for 15 min to achieve complete dissolution. The pH of the prepared derivatization reagents was measured in triplicates for all reaction conditions after ten times dilution in UP water using narrow range pH indicator strips. For the native conditions, the pH of all reagents was approx. 6. For all catalysts, the reaction space was expanded to acidic and basic pH. Acidic conditions were achieved by the addition of 1 µL 10× diluted 37% HCl (1.21 M) to 20 µL derivatization reagent, resulting in a final concentration of 0.058 M (resulting in approx. pH 3), while basic conditions were accomplished by the addition of 1 µL 3 M NaOH to 20 µL derivatization reagent to final concentration of 0.143 M (resulting in approx. pH 9). The addition of acidic or basic modifier was due right before sample application.

### 4.4. N-glycan Release

*N*-glycans from human plasma were released in acidic PBS (pH 5.6), as described before [24]. In short, 20 µL 2% SDS was added to 10 µL plasma, shaken for 5 min on a horizontal shaking platform at 1350 rpm and incubated for 10 min at 60 °C. The sample was allowed to come to room temperature before the addition of 20 µL release mixture. The sample was shaken for 5 min at 1350 rpm, followed by overnight incubation at 37 °C and used for sialic acid derivatization without further processing steps.

### 4.5. Sialic Acid Derivatization

Linkage-specific sialic acid derivatization in ethanol using one of the catalysts was performed according to established procedures [13]. Briefly, 20 µL of HOBt + EDC, 6-Cl-HOBt + EDC, + 6-CF_3_-HOBt + EDC, or HOAt + EDC in ethanol was added to the wells of a 96-well NUNC V-bottom plate (Thermo Scientific, Waltham, MA, USA). For non-native samples, 1 µL of acidic (3.7% HCl) or basic (3M NaOH) modifier was added right before sample application. Sialic acid derivatization was performed on either 1 µL of one of the SL standards (100 µg) or 1 µL released *N*-glycans mixture. After adding the samples, the reaction mixtures were incubated for 1 h at 37 °C or 60 °C, followed by cotton-hydrophilic interaction liquid chromatography (HILIC) solid phase extraction (SPE) and MALDI-TOF-MS analysis. All samples were prepared in triplicate.

### 4.6. Cotton-HILIC SPE

Cotton-HILIC SPE of derivatized SL and released *N*-glycans was performed using in-house prepared microtips [13,43] after adding 20 µL ACN directly to the reagents. Microtips were prepared by packing 3 mm cotton thread (approx. 180 μg, Pipoos, Utrecht, The Netherlands) into 20 µL pipette tips (Rainin Instrument, Oakland, CA, USA) using tweezers, followed by the application of 50 kPa air pressure difference [13] (Figure S3). Prior to enrichment, cotton tips were washed three times with 15 µL UP water, conditioned three times with 15 µL 85% ACN followed by sample loading (twenty times 15 µL). The cotton tips were then washed three times with 15 µL 85% ACN 0.1% TFA and three times with 15 µL 85% ACN. Finally, the retained glycans were eluted in 10 µL UP water.

### 4.7. MALDI-TOF-MS Analysis

MALDI-TOF-MS analyses were performed on an Ultraflex II mass spectrometer (Bruker Daltonics, Bremen, Germany) equipped with a Smartbeam laser. Spectra were acquired in reflectron positive mode collecting a total of 10,000 laser shots at a laser frequency of 1000 Hz, using 25 kV acceleration voltage. Prior to measurement, the instrument was calibrated with peptide calibration mix II (Bruker Daltonics). A *m*/*z* window of *m*/*z* 500–1000 was used for SL standards, and of *m*/*z* 1000–5000 for released *N*-glycans.

To prepare the glycan samples for MALDI, 1 µL 2,5-DHB (5 mg/mL in 50% ACN with 1 mM NaOH) was spotted on an AnchorChip 800/384 TF MALDI target (Bruker Daltonics), 1 µL HILIC enriched glycans was added and the spots were left to dry by air. Eventually, the dried samples were re-crystallized with the addition of 1 µL ethanol in order to obtain a microcrystalline to amorphous appearance and increased shot-to-shot repeatability.

### 4.8. Data Analysis

Data analysis was performed with the in-house developed software MassyTools [44], designed for high-throughput, targeted data extraction, and quality control of MALDI-MS data. Internal calibration was performed based on a selected calibrant list (Table S6) (Table S7), followed by targeted data extraction using predefined glycan compositions. Data analysis was performed based on quality control calculations such as isotopic pattern quality (IPQ), ppm, and S/N. Total area normalization of the extracted glycan signals passing quality control criteria was performed for each spectrum (Table S2) (Table S5). To evaluate linkage specificity over the catalysts, di- and triantennary species were total area normalized to 100% separately (Figure 5B) (Table S5). HOBt under native reaction conditions was used as a benchmark to other catalysts. Averages and SDs were calculated from triplicate measurements using Microsoft Excel.

## 5. Patents

The work described in this paper builds on the patent “Glycan Analysis by Derivatizing Sialic Acid Residues” which has been granted by the United States Patent and Trademark Office (USPTO; patent number 10233262) and the European Patent Office (EP3071601).

## Supplementary Materials

The following are available online, Table S1: Information on physico-chemical characteristics, toxicity and availability of the used catalysts; Table S2: Linkage-specificity of catalysts by using sialyllactose standards; Table S3: Signal-to-Noise ratios (S/N) for each tested reaction condition; Table S4: List and overview of detected glycans using 6-Cl-HOBt at native conditions; Table S5: Evaluating the linkage-specificity of the reactions using the catalysts for complex samples; Table S6: Calibration masses list for sialyllactose standards; Table S7: Calibration masses list for total plasma released *N*-glycome; Figure S1: MALDI-TOF-MS spectrum of amidated 2,6-sialyllactose standard when using Oxyma Pure as catalyst at native condition; Figure S2: Signal-to-Noise ratio of ethyl esterified, lactonized and unmodified ([M + Na]+ and [M − H + 2Na]+) reaction products on α2,6 (A, B) and α2,3-sialyllactose (C, D) at 60 (A, C) and 37 °C (B, D) incubation.; Figure S3: Representative picture of an in-house prepared cotton-HILIC SPE tip.

## References

[1] Moremen K.W., Tiemeyer M., Nairn A.V. (2012). Vertebrate protein glycosylation: Diversity, synthesis and function. Nat. Rev. Mol. Cell Biol..

[2] Varki A. (2017). Biological roles of glycans. Glycobiology.

[3] Kim Y.J., Varki A. (1997). Perspectives on the significance of altered glycosylation of glycoproteins in cancer. Glycoconj. J..

[4] Khoury G.A., Baliban R.C., Floudas C.A. (2011). Proteome-wide post-translational modification statistics: Frequency analysis and curation of the swiss-prot database. Sci. Rep..

[5] Stowell S.R., Ju T., Cummings R.D. (2015). Protein glycosylation in cancer. Annu. Rev. Pathol..

[6] Varki A. (2008). Sialic acids in human health and disease. Trends Mol. Med..

[7] Hedlund M., Ng E., Varki A., Varki N.M. (2008). alpha 2-6-Linked sialic acids on *N*-glycans modulate carcinoma differentiation in vivo. Cancer Res..

[8] Kang P., Mechref Y., Klouckova I., Novotny M.V. (2005). Solid-phase permethylation of glycans for mass spectrometric analysis. Rapid Commun. Mass Spectrom..

[9] Harvey D.J. (2009). Analysis of carbohydrates and glycoconjugates by matrix-assisted laser desorption/ionization mass spectrometry: An update for 2003-2004. Mass Spectrom. Rev..

[10] Powell A.K., Harvey D.J. (1996). Stabilization of sialic acids in N-linked oligosaccharides and gangliosides for analysis by positive ion matrix-assisted laser desorption ionization mass spectrometry. Rapid Commun. Mass Spectrom..

[11] Selman M.H., Hoffmann M., Zauner G., McDonnell L.A., Balog C.I., Rapp E., Deelder A.M., Wuhrer M. (2012). MALDI-TOF-MS analysis of sialylated glycans and glycopeptides using 4-chloro-alpha-cyanocinnamic acid matrix. Proteomics.

[12] Reiding K.R., Hipgrave Ederveen A.L., Rombouts Y., Wuhrer M. (2016). Murine Plasma N-Glycosylation Traits Associated with Sex and Strain. J. Proteome Res..

[13] Reiding K.R., Blank D., Kuijper D.M., Deelder A.M., Wuhrer M. (2014). High-throughput profiling of protein N-glycosylation by MALDI-TOF-MS employing linkage-specific sialic acid esterification. Anal. Chem..

[14] Wheeler S.F., Domann P., Harvey D.J. (2009). Derivatization of sialic acids for stabilization in matrix-assisted laser desorption/ionization mass spectrometry and concomitant differentiation of alpha(2 --> 3)- and alpha(2 --> 6)-isomers. Rapid Commun. Mass Spectrom..

[15] El-Faham A., Albericio F. (2011). Peptide coupling reagents, more than a letter soup. Chem. Rev..

[16] Valeur E., Bradley M. (2009). Amide bond formation: Beyond the myth of coupling reagents. Chem. Soc. Rev..

[17] Liu X., Qiu H., Lee R.K., Chen W., Li J. (2010). Methylamidation for sialoglycomics by MALDI-MS: A facile derivatization strategy for both alpha2,3- and alpha2,6-linked sialic acids. Anal. Chem..

[18] Li H., Gao W., Feng X., Liu B.F., Liu X. (2016). MALDI-MS analysis of sialylated *N*-glycan linkage isomers using solid-phase two step derivatization method. Anal. Chim. Acta.

[19] Nishikaze T., Tsumoto H., Sekiya S., Iwamoto S., Miura Y., Tanaka K. (2017). Differentiation of Sialyl Linkage Isomers by One-Pot Sialic Acid Derivatization for Mass Spectrometry-Based Glycan Profiling. Anal. Chem..

[20] Yang S., Zhang L., Thomas S., Hu Y., Li S., Cipollo J., Zhang H. (2017). Modification of Sialic Acids on Solid Phase: Accurate Characterization of Protein Sialylation. Anal. Chem..

[21] Hanamatsu H., Nishikaze T., Miura N., Piao J., Okada K., Sekiya S., Iwamoto S., Sakamoto N., Tanaka K., Furukawa J.I. (2018). Sialic Acid Linkage Specific Derivatization of Glycosphingolipid Glycans by Ring-Opening Aminolysis of Lactones. Anal. Chem..

[22] Suzuki N., Abe T., Natsuka S. (2019). Quantitative LC-MS and MS/MS analysis of sialylated glycans modified by linkage-specific alkylamidation. Anal. Biochem..

[23] Yang S., Wu W.W., Shen R.F., Bern M., Cipollo J. (2018). Identification of Sialic Acid Linkages on Intact Glycopeptides via Differential Chemical Modification Using IntactGIG-HILIC. J. Am. Soc. Mass Spectrom..

[24] Vreeker G.C.M., Nicolardi S., Bladergroen M.R., van der Plas C.J., Mesker W.E., Tollenaar R., van der Burgt Y.E.M., Wuhrer M. (2018). Automated Plasma Glycomics with Linkage-Specific Sialic Acid Esterification and Ultrahigh Resolution MS. Anal. Chem..

[25] Klein A. (2008). Human total serum N-glycome. Adv. Clin. Chem..

[26] Clerc F., Reiding K.R., Jansen B.C., Kammeijer G.S., Bondt A., Wuhrer M. (2016). Human plasma protein N-glycosylation. Glycoconj. J..

[27] Yang S., Jankowska E., Kosikova M., Xie H., Cipollo J. (2017). Solid-Phase Chemical Modification for Sialic Acid Linkage Analysis: Application to Glycoproteins of Host Cells Used in Influenza Virus Propagation. Anal. Chem..

[28] Lageveen-Kammeijer G.S.M., de Haan N., Mohaupt P., Wagt S., Filius M., Nouta J., Falck D., Wuhrer M. (2019). Highly sensitive CE-ESI-MS analysis of *N*-glycans from complex biological samples. Nat. Commun..

[29] Miura Y., Shinohara Y., Furukawa J., Nagahori N., Nishimura S. (2007). Rapid and simple solid-phase esterification of sialic acid residues for quantitative glycomics by mass spectrometry. Chemistry.

[30] Schwedler C., Kaup M., Petzold D., Hoppe B., Braicu E.I., Sehouli J., Ehlers M., Berger M., Tauber R., Blanchard V. (2014). Sialic acid methylation refines capillary electrophoresis laser-induced fluorescence analyses of immunoglobulin GN-glycans of ovarian cancer patients. Electrophoresis.

[31] Mitra I., Snyder C.M., Zhou X., Campos M.I., Alley W.R., Novotny M.V., Jacobson S.C. (2016). Structural Characterization of Serum *N*-Glycans by Methylamidation, Fluorescent Labeling, and Analysis by Microchip Electrophoresis. Anal. Chem..

[32] Snyder C.M., Zhou X., Karty J.A., Fonslow B.R., Novotny M.V., Jacobson S.C. (2017). Capillary electrophoresis-mass spectrometry for direct structural identification of serum *N*-glycans. J. Chromatogr. A.

[33] Dedova T., Braicu E.I., Sehouli J., Blanchard V. (2019). Sialic Acid Linkage Analysis Refines the Diagnosis of Ovarian Cancer. Front. Oncol..

[34] De Haan N., Reiding K.R., Haberger M., Reusch D., Falck D., Wuhrer M. (2015). Linkage-specific sialic acid derivatization for MALDI-TOF-MS profiling of IgG glycopeptides. Anal. Chem..

[35] Shubhakar A., Reiding K.R., Gardner R.A., Spencer D.I.R., Fernandes D.L., Wuhrer M. (2014). High-Throughput Analysis and Automation for Glycomics Studies. Chromatographia.

[36] Carpino L.A. (1993). 1-Hydroxy-7-azabenzotriazole. An efficient peptide coupling additive. J. Am. Chem. Soc..

[37] El-Faham A., Albericio F. (2009). Synthesis and Application ofN-Hydroxylamine Derivatives as Potential Replacements for HOBt. Eur. J. Org. Chem..

[38] Jiang L., Davison A., Tennant G., Ramage R. (1998). Synthesis and application of a novel coupling reagent, ethyl 1-hydroxy-1H -1,2,3-triazole-4-carboxylate. Tetrahedron.

[39] Subiros-Funosas R., Prohens R., Barbas R., El-Faham A., Albericio F. (2009). Oxyma: An efficient additive for peptide synthesis to replace the benzotriazole-based HOBt and HOAt with a lower risk of explosion. Chemistry.

[40] Wehrstedt K.D., Wandrey P.A., Heitkamp D. (2005). Explosive properties of 1-hydroxybenzotriazoles. J. Hazard Mater..

[41] Schauer R. (1978). Characterization of sialic acids. Methods Enzymol.

[42] Holst S., Deuss A.J.M., van Pelt G.W., van Vliet S.J., Garcia-Vallejo J.J., Koeleman C.A.M., Deelder A.M., Mesker W.E., Tollenaar R.A., Rombouts Y. (2016). N-glycosylation Profiling of Colorectal Cancer Cell Lines Reveals Association of Fucosylation with Differentiation and Caudal Type Homebox 1 (CDX1)/Villin mRNA Expression. Mol. Cell. Proteom..

[43] Selman M.H.J., Hemayatkar M., Deelder A.M., Wuhrer M. (2011). Cotton HILIC SPE Microtips for Microscale Purification and Enrichment of Glycans and Glycopeptides. Anal. Chem..

[44] Jansen B.C., Reiding K.R., Bondt A., Hipgrave Ederveen A.L., Palmblad M., Falck D., Wuhrer M. (2015). MassyTools: A High-Throughput Targeted Data Processing Tool for Relative Quantitation and Quality Control Developed for Glycomic and Glycoproteomic MALDI-MS. J. Proteom. Res..

