# Supplementary material for: Expanding the Reaction Space of Linkage-Specific Sialic Acid Derivatization
Source: Molecules. 2019 Oct 8;24(19):3617. doi: 10.3390/molecules24193617 (PMC6803949; doi:10.3390/molecules24193617)
Supplement: Supplementary file 1 [file molecules-24-03617-s001.zip › Supplementary Figures_Image quality changed.docx]

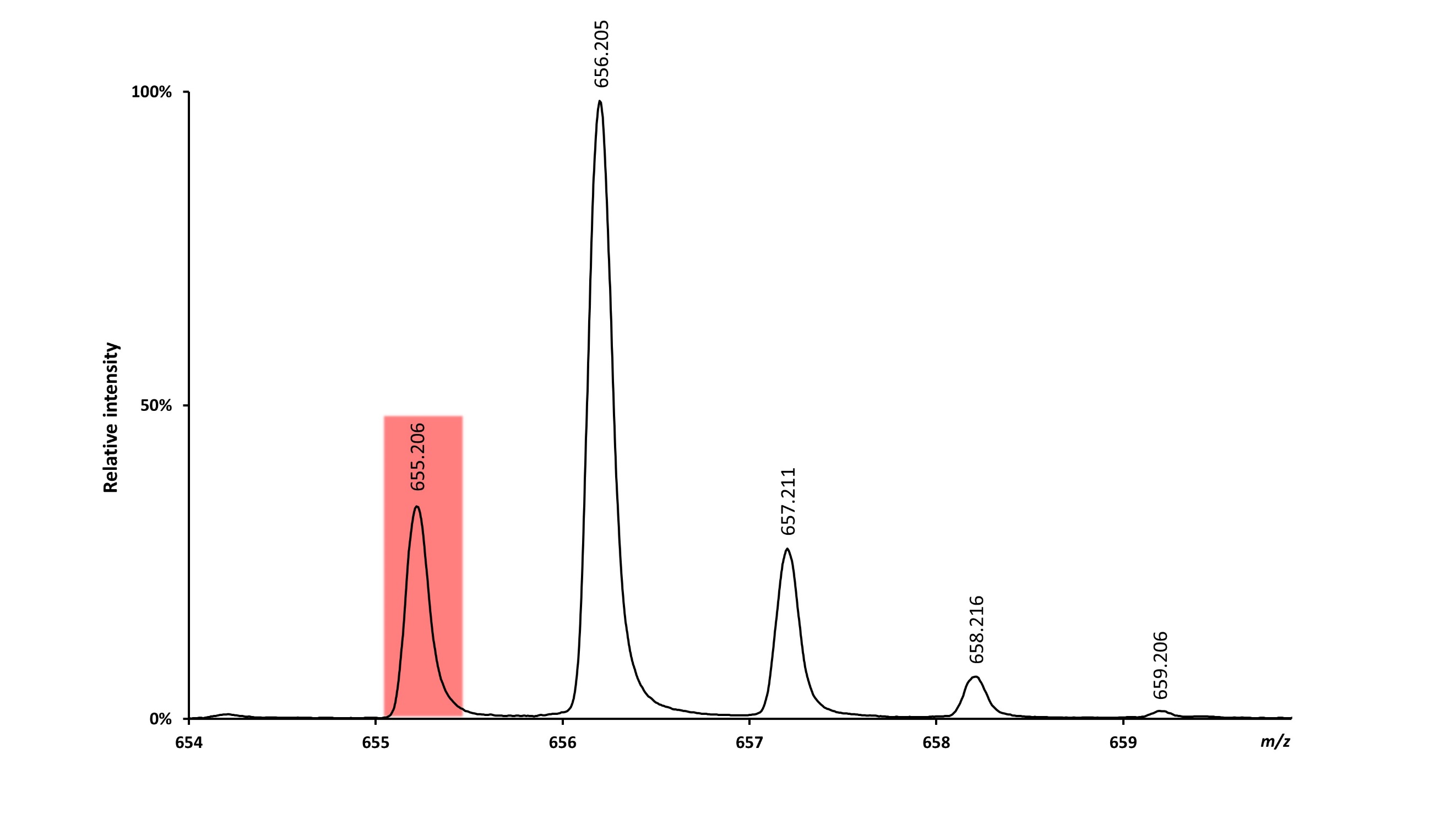


**Figure S1.** MALDI-TOF-MS spectrum of amidated 2,6-sialyllactose standard when using Oxyma Pure as catalyst at native condition. Shown in red is the amidated standard ([M+Na]^+^ = 655.217), that overlapped with the under modified sialyllactose standard ([M+Na]^+^ = 656.201).


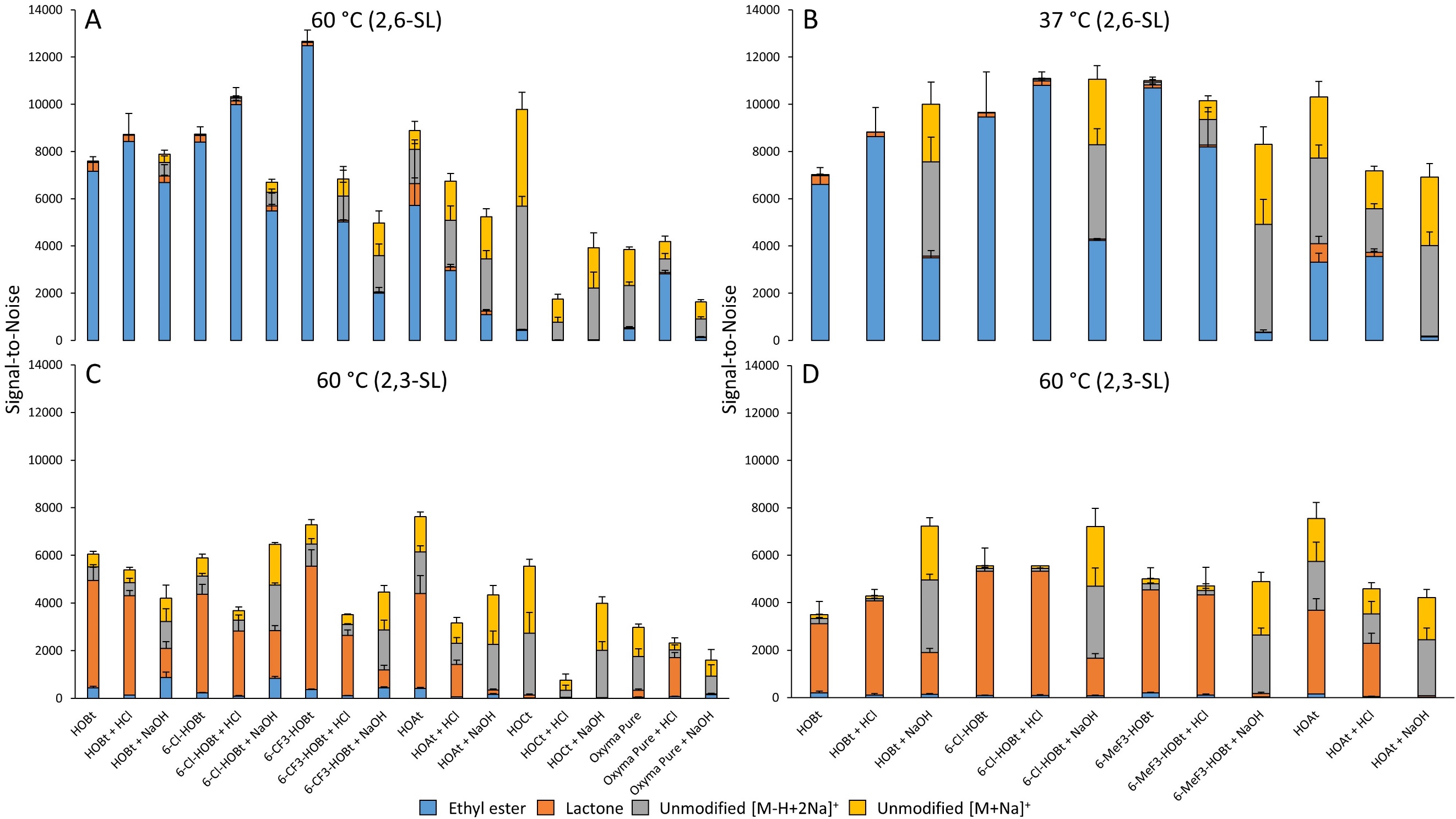


**Figure S2.** Signal-to-Noise ratio of ethyl esterified, lactonized and unmodified ([M+Na]^+^ and [M-H+2Na]^+^) reaction products on α2,6 (**A**, **B**) and α2,3-sialyllactose (**C**, **D**) at 60 (**A**, **C**) and 37 °C (**B**, **D**) incubation.

**
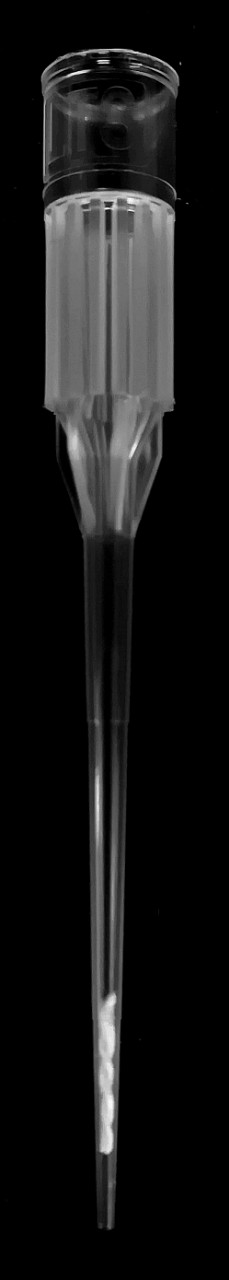
**

**Figure S3.** Representative picture of an in-house prepared cotton-HILIC SPE tip.
